# Editorial: Role of non-coding RNAs, metabolites, and extracellular vesicles in disease regulation and health

**DOI:** 10.3389/fgene.2023.1206569

**Published:** 2023-06-09

**Authors:** Olanrewaju B. Morenikeji, Naseer A. Kutchy

**Affiliations:** ^1^ Division of Biological and Health Sciences, University of Pittsburgh at Bradford, Bradford, PA, United States; ^2^ Department of Animal Sciences, Rutgers, The State University of New Jersey, New Brunswick, NJ, United States

**Keywords:** non-coding RNAs, extracellular vesicles, disease, gene regulation, health

Non-coding ribonucleic acids (small or large ncRNAs) are involved in epigenetic regulations and could mediate pre- or post-transcriptional gene silencing. These molecules are opening new avenues for therapeutic purposes with putative consequences for immunity. Similarly, emerging studies have demonstrated that extracellular vesicles (EVs) are involved in many immune response dysregulations and diseases. EVs, such as apoptotic bodies, exosomes, and microvesicles, are membrane-bound vesicles ranging in size from 30 to 1,000 nm in diameter. During pathological conditions, the number, size, and content of EVs are found to be altered and have been shown to play crucial roles in disease progression. Likewise, bacteria or a host can produce EVs containing ncRNAs that can mediate intercellular communication with epithelial and immune cells, potentially regulating the expression of genes involved in resistance to pathologies.

This Research Topic brought together various articles, including primary research articles, reviews, methods, and systematic reviews, that focus on elucidating the role of ncRNAs and EVs in molecular immunotherapy and cross talk with genetic or epigenetic mechanisms in controlling cellular function in disease biology as potential therapeutic tools.

Nie et al. discussed the lack of early diagnosis of hepatocellular carcinoma (HCC), which results in poor prognosis for patients. The authors mentioned that early HCC diagnosis currently depends on the level of serum alpha-fetoprotein (AFP) and imaging technologies which are not optimal. Through meta-analysis, Nie et al. identified key circular RNAs in exosomes for screening and diagnosing HCC. According to the authors, HCC is rated the fourth highest cause of death worldwide but lacks accurate biomarkers for early detection. The authors emphasized the role of the unique expression of circular RNAs in HCC including circ_0000798, circSATD3, hsa_circ_0007750, and cMTO1 in suppressing HCC progression with their possible use in diagnosis or treatment.

Yousuf et al. used a panel of three miRNAs (miR-221, miR-222, and Let-7a) to detect HCC in the early stage. The authors reported a correlation between the expression of the three miRNAs and AFP in the serum and tissues, indicating promises for early diagnosis of HCC. Therefore, they proposed the use of these miRNAs in combination with AFP as biomarkers for early diagnosis of HCC and on a large cohort of HCC patients. Lu et al.’s study is similar to that of Yousuf et al. because it identified the use of miRNAs for the diagnosis and prognosis of HCC. However, the fundamental difference was that Lu et al.’s study was based on a meta-analysis. The authors cataloged different studies from PubMed, Embase, and Wan Fang databases and identified the miR-17–92 cluster in context of diagnosis and prognosis of HCC. Lu et al. determined the sensitivity, specificity, and diagnostic odds ratio (DOR) on the miR-17–92 cluster for disease diagnosis. They found that the sensitivity of the diagnosis of HCC using the miR-17–92 cluster was 0.75 [at 95% confidence interval (CI)], with a specificity of 0.73 at 95% CI and a DOR of 7.87. Based on their results, Lu et al. finally suggested that the miR-17–92 cluster could be used as a diagnostic and prognostic biomarker for HCC.

A mini-review by Ruiz-Manriquez et al. discussed miRNA-mediated regulation in key signaling pathways during hepatocellular carcinoma development. The authors emphasized that microRNAs are directly or indirectly involved in cellular signaling, and manipulating those miRNAs can potentially affect key signaling pathways resulting in carcinogenesis. In addition, Ruiz-Manriquez et al. suggested that miRNA-media/ted PI3K/Akt/mTOR, Hippo-YAP/TAZ, and Wnt/β-catenin signaling pathways could lead to HCC development. This perspective could help for a better understanding of diagnosis and management of HCC.

Knowing that long non-coding RNAs (lncRNAs) could act as epigenetic regulators in diverse cellular processes including ferroptosis and iron metabolism, Hong-Bin et al. used RNA sequencing analysis and clinical information to decipher an iron metabolic-related lncRNA signature that could be used to determine osteosarcoma (OS) survival and the immune landscape. The authors identified a total of 302 iron metabolism-related lncRNAs including lncRNA GAS5, UNC5B-AS1, PARD6G-AS1, and LINC01060 that are capable of regulating cell proliferation, migration, and invasion during osteosarcoma. The authors concluded that iron metabolism-related lncRNA signatures had good performance in predicting survival outcomes and the immune landscape for OS patients.

Zhao et al. showed the importance of 11 autophagy-related lncRNAs for esophageal squamous cell carcinoma (ESCC). They analyzed the immune function and tumor mutational burden between two groups with varying sensitivity to chemotherapy and immunotherapy. Overall, Zhao et al. reported the signature expression of some lncRNAs, including AL078604.2, BDNF-AS, HAND2-AS1, and ZEB1-AS1, among others, to be co-expressed with autophagy genes. Importantly, the authors provided an lncRNA risk model that could be used as a tool for the prognosis and treatment of patients with ESCC.

As described by Peeples et al., neonatal hypoxic–ischemic brain injury (HIBI) is reported to present varying phenotypes, posing difficult diagnosis and increased morbidity and mortality despite available therapy. This calls for an alternative novel therapy to safeguard the life of infants. Peeples et al. elucidated the role of miRNAs in gene regulation during HIBI as a promising therapeutic target for miRNA-based therapy. Overall, they found 61 unique miRNAs being differentially expressed in various regions of the brain including miR-410-5p, -1264-3p, 1298-5p, -5,126, and -34b-3p. These miRNAs targeted pathways associated with inflammation, metabolism, and cell death.

Zhang et al. highlighted the importance of miRNAs in coronary artery disease (CAD) from microarray datasets using various bioinformatics tools. The authors defined CAD as a cardiovascular disease associated with low-density lipoprotein cholesterol and categorized it as a major source of death worldwide. They also observed a correlation between miRNA expression pattern and CAD, although the role of many of the miRNAs in CAD is still unclear. Zhang et al. reported that miR-22-3p interacted with eight transcription factors in the cardiovascular system and suggested it as a better predictor for CAD, making it a potential biomarker for disease diagnosis.

It is noteworthy that host ncRNAs also play a significant role in modulating viral replication during host–pathogen interactomes. Morenikeji et al. identified important miRNAs that could target RNA-dependent RNA polymerase (RdRp), an enzyme necessary for coronavirus replication inside the host. Due to frequent mutations in coronavirus, several disease control measures, such as vaccines and antiviral drugs, are less effective in completely controlling viral replication. Since the RdRp is conserved among coronavirus variants, the authors found it to be an important target for miRNAs in controlling virus replication. Ultimately, the authors reported the ability of hsa-miR-1283, hsa-miR-579-3p, and hsa-miR-664b-3p to block virus replication through binding to their RdRp target and noted their potential use as a broad-spectrum antiviral.

Li et al. shed light on the role of melatonin and its interactions with ncRNAs in regulating osteoarthritis (OA). OA is a slowly progressive and irreversible disease of joints and lacks early treatments. Therefore, the authors gathered substantial knowledge about the melatonin-mediated modulation of ncRNAs in the early stage of OA. In addition, the authors proposed that the interaction between ncRNAs and target genes constitutes a complex network of OA regulation. In conclusion, they suggested that a combination of ncRNAs or their regulators with melatonin may be a new approach for the treatment of OA in the future.

A review by Liu et al. examined the role of circRNA E3 ubiquitin–protein ligase (circRNA ITCH) in clinical applications during tumor and non-tumor diseases. circRNA is a closed-loop RNA capable of up- or down-regulating gene expression at different levels. They have been implicated in cellular processes, including inhibiting cell proliferation, migration, and invasion and promoting apoptosis. Due to their molecular function and other involvement in biological processes, circ-ITCH could be used as a marker to monitor disease progression.

The uterine environment is important for a successful pregnancy, especially during the peri-implantation stage. Hitit et al. highlighted the role of miRNAs during embryo implantation. They also provided an understanding of circulating miRNA in maternal plasma as a biomarker for early detection of pregnancy in sheep. They found that 22 miRNA expression patterns were associated with the estrous cycle and early pregnancy, of which two were oar-miR-218a- and oar-miR-1185-3p-targeted genes related to embryonic morphogenesis and developmental process. The authors concluded that circulating miRNAs from plasma hold a promise for early detection of pregnancy in sheep.

Body fluids carrying small extracellular vesicles (sEVs) have been isolated from blood, pleural fluids, saliva, and urine as biomarkers. These sEVs could be carrying cargoes with exosomal miRNAs (exomiRs), which have been found responsible for cancer pathogenesis. Some exomiR expressions could indicate cancer progression, cancer growth, and drug response/resistance. Furthermore, Alotabi described the use of exomiRs as biomarkers for cancer diagnosis, treatment response, metastasis, and promotion of T cell anti-tumor immunity via activation of immune checkpoint molecules. The author pinpointed how exomiRs could better regulate the expression of immune checkpoint molecules, leading to activation of T cell anti-tumor immunity. Alotabi reviewed multiple literature records to underscore the importance of sEVs, especially in patients with immune rejection.

The article by Luo et al. also discussed the role of sEVs carrying ncRNA cargoes and summarized their impact on kidney diseases. The authors found that sEVs could be produced by all cell types in the kidney, including neighboring cells like renal tubular epithelial cells, podocytes, collecting duct cells, and leap cells. These sEVs could then be loaded with aberrant ncRNAs that could be responsible for many kidney diseases. Luo et al. concluded that the sEV-ncRNAs could play a fundamental role in the early detection and prognosis of kidney diseases.

Singh et al. identified estrus biomarkers through biofluid, such as saliva, using proteomic profiling of buffaloes in different stages of the estrus cycle. They used both label-free quantitation (LFQ) and labeled (TMT) quantitation and mass spectrometry analysis. The authors reported an over-expression of proteins such as SERPINB1, HSPA1A, VMO1, SDF4, LCN1, OBP, and ENO3 in buffalo saliva estrus, depicting a novel method for estrus detection. The markers were functionally classified to be over-represented in several ontologies, including glycolysis, pyruvate metabolism, endopeptidase inhibitor activity, salivary secretion, innate immune response, calcium–ion binding, oocyte meiosis, and estrogen signaling. Singh et al. concluded by generating an array panel of candidate proteins that may be considered buffalo estrus biomarkers, applied in the development of a diagnostic kit for estrus detection in buffaloes.

Finally, our editorial summarizes various articles on the role of non-coding ribonucleic acids and extracellular vesicles to understand epigenetic mechanisms that control cellular functions in different diseases. We believe that various findings in this research topic would be of much help in expanding the basic knowledge in disease studies and as potential therapeutic tools. We appreciate all contributors to this research article, including the authors, reviewers, and the editorial team, Frontiers in Genetics.

